# Albumin to Total Cholesterol Ratio and Mortality in Peritoneal Dialysis

**DOI:** 10.3389/fmed.2022.896443

**Published:** 2022-06-09

**Authors:** Xianfeng Wu, Jiao Meng, Lei Zhou, Xiaojiang Zhan, Yueqiang Wen, Xiaoyang Wang, Xiaoran Feng, Niansong Wang, Fenfen Peng, Junnan Wu

**Affiliations:** ^1^Department of Nephrology, Shanghai Jiao Tong University Affiliated Sixth People’s Hospital, Shanghai, China; ^2^Clinical Research Center for Chronic Kidney Disease, Shanghai Jiao Tong University Affiliated Sixth People’s Hospital, Shanghai, China; ^3^Department of Nephrology, Zhejiang University Medical College Affiliated Sir Run Run Shaw Hospital, Hangzhou, China; ^4^Evergreen Tree Nephrology Association, Guangzhou, China; ^5^Department of Nephrology, The First Affiliated Hospital of Nanchang University, Nanchang, China; ^6^Department of Nephrology, The Second Affiliated Hospital of Guangzhou Medical University, Guangzhou, China; ^7^Department of Nephrology, The First Affiliated Hospital of Zhengzhou University, Zhengzhou, China; ^8^Department of Nephrology, Jiujiang No. 1 People’s Hospital, Jiujiang, China; ^9^Department of Nephrology, Zhujiang Hospital of Southern Medical University, Guangzhou, China

**Keywords:** albumin to total cholesterol ratio, mortality, peritoneal dialysis, cardiovascular, prognosis

## Abstract

**Background:**

Serum albumin and total cholesterol are associated with mortality. In clinical practice, evaluating the association of combining album and total cholesterol with mortality may be more reasonable. Thus, we examined the association between serum albumin to total cholesterol ratio and mortality in peritoneal dialysis (PD) patients.

**Methods:**

We conducted a retrospective cohort study of 3447 incident continuous ambulatory peritoneal dialysis (CAPD) patients from five PD centers in China from 1 January 2005 and 31 May 2020. The association between albumin to total cholesterol ratio and mortality was evaluated.

**Results:**

With a median follow-up of 39.3 months, 762 (22.1%) all-cause deaths occurred, including 382 (11.1%) cardiovascular deaths. As compared with a serum albumin to total cholesterol ratio of 0.77–0.82 (reference range), a higher ratio (>0.82) was associated with increased risks of all-cause mortality[hazards ratio (HR), 1.54; 95% confidence interval (CI), 1.16–2.05, *E*-value = 2.45] and cardiovascular mortality (HR, 2.10; 95% CI, 1.35–3.29, *E*-value = 3.62). A lower ratio (<0.77) was also associated with increased risks of all-cause mortality (HR, 1.46; 95% CI, 1.10–1.94, *E*-value = 2.28) and cardiovascular mortality (HR, 1.78; 95% CI, 1.14–2.78, *E*-value = 2.96) compared with the reference. No interaction was observed in subgroup analyses of age, sex, diabetes mellitus, hypertension, prior cardiovascular disease, and hyperlipidemia, and malnutrition (serum albumin <3.6 g/dL).

**Conclusion:**

An albumin to total cholesterol ratio before the start of PD between 0.77 and 0.82 was associated with a lower risk of death than a higher or lower ratio, resulting in a U-curve association. Therefore, serum albumin to total cholesterol ratio, as an inexpensive and readily available biochemical biomarker, may further improve the stratification risk of mortality in PD patients.

## Introduction

Moderate to severe malnutrition is associated with an increased risk of death in peritoneal dialysis (PD) patients. Early publications estimated that 40–66% of PD patients in the United States are malnourished (1–6). In a subsequent study that defined protein-energy wasting by serum albumin of <3.8 g/dL, 63% of PD patients were classified as malnourished (7). Many patients are malnourished when they begin dialysis. As an example, in one study, approximately 75% of patients initiating PD were malnourished (8). Thus, lower serum albumin is very common in PD patients.

Peritoneal dialysis patients typically have elevated levels of serum total cholesterol, whereas hemodialysis patients have normal or low levels of serum total cholesterol (9). As PD patients have different lipid profiles, traditional lipid profiles may have a different association with mortality in PD patients. As an example, a strategy to reduce low density lipoprotein (LDL) demonstrated no beneficial effect on cardiovascular disease in dialysis patients (10). Therefore, it may not be appropriate to evaluate the risk of mortality using only traditional lipid profiles in PD patients.

Previous studies have reported that lower serum albumin is associated with a higher risk of mortality (11–14), and lower cholesterol levels have been consistently associated with a higher risk of mortality in dialysis patients (14–17). Notably, many individuals who start PD have lower serum albumin and higher total cholesterol levels in clinical practice. Thus, based on serum albumin or total cholesterol levels, it is difficult to speculate the prognosis of dialysis patients. Hence, it is mandatory to combine serum albumin with total cholesterol to evaluate the prognosis in PD patients. An early study reported that the total cholesterol to albumin ratio could be used as an alternative parameter in predicting the risk of cardiovascular disease in the general population (18). However, there is no study to date examining the association between albumin to total cholesterol ratio and mortality in dialysis patients. Our study aimed to evaluate the association between albumin to total cholesterol ratio and mortality in patients on continuous ambulatory peritoneal dialysis (CAPD).

## Materials and Methods

### Study Design and Participants

We conducted a retrospective study that included 3566 incident CAPD patients from five PD centers in China between 1 January 2005 and 31 May 2020. To evaluate the association between albumin to total cholesterol 1 week before the start of PD and mortality in the real-world setting, we only excluded patients aged <18 years and those with <3 months of the follow-up. the data were de-identified, and the need for informed consent was waived. The study protocol complied with the Declaration of Helsinki and had full approval from each Clinical Research Ethics Committee.

### Follow Up and Data Collection

Patients were advised to initiate PD with professional and clinical evaluation from nephrologists. In all patients, thorough medical records were reviewed by trained nurses in each dialysis center at study entry. In China, the patient must receive the first dialysis in the hospital, suggesting most of the patient’s data can be obtained within 1 week before the first dialysis. Thus, we defined baseline as 1 week (5.3 ± 1.2 days) before the first PD. All laboratory parameters from fasting blood samples were measured in each tertiary hospital’s laboratory department. Specifically, we included age at study entry, sex, body mass index, current smoker, current alcohol use, systolic blood pressure, diastolic blood pressure, comorbidities [diabetes mellitus, hypertension, prior cardiovascular disease, hyperlipidemia, chronic obstructive pulmonary disease (COPD), gastrointestinal bleeding], medication use [calcium antagonist, beta-blockers, angiotensin-converting enzyme inhibitors/angiotensin II receptor blockers (ACEI or ARBs), diuretics, and statins], and laboratory measurements [hemoglobin, albumin, estimated glomerular filtration rate (eGFR), total cholesterol, high density lipoprotein (HDL), and LDL]. There was no exposure to all patients with any intervention. Patients needed to return to each center at least quarterly for an overall medical assessment. The trained nurses conducted monthly face-to-face interviews or monthly telephone interviews to assess their general condition and related medications. All patients received CAPD treatment. Conventional dialysis solutions (Dianeal 1.5, 2.5, or 4.25% dextrose; Baxter Healthcare, Guangzhou, China), Y sets, and twin bag systems were used in all CAPD patients. No patients received automated PD.

### Outcome Measurements

Our primary outcome measure was all-cause and cardiovascular mortality. We determined death causes based on medical files of admission. If patients died out of hospitals, we determined death causes according to interviewing with family members by telephone to acknowledge death’s circumstances, combining with information from medical records of PD centers. Details for the CAPD follow-up were previously described elsewhere (19). The follow-up period was from the start of PD to the date of death, transfer to hemodialysis, receiving renal transplantation, transfer to other dialysis centers, loss of follow-up, or 31 May 2020. Patients who were lost to follow-up were censored at the date of the last examination.

### Statistical Analysis

Differences in the baseline characteristics among the study population in the different categories of albumin to total cholesterol ratio were compared using the Chi-square test for categorical variables and analysis of variance for continuous variables. We used restricted-cubic-spline plots to explore the shape of the association between albumin to total cholesterol ratio and mortality, fitting a restricted-cubic-spline function with four knots (at the 25th, 50th, 75th, and 95th percentiles) (20).

Based on our restricted-cubic-spline plots for the primary outcome, we selected a level of 0.77–0.82 as the reference category for the albumin to total cholesterol ratio. Cumulative all-cause and cardiovascular mortality were analyzed using the Kaplan–Meier failure function. Using four sequential models, we performed a multivariable Cox proportional hazards model to determine the association between albumin to total cholesterol ratio and all-cause and cardiovascular mortality. Model 1 was adjusted for age, sex, body mass index, current smoker, current alcohol use, and systolic blood pressure. Model 2 included model 1 and diabetes mellitus, hypertension, prior cardiovascular disease, COPD, gastrointestinal bleeding, and hyperlipidemia. Model 3 included model 2 and taking calcium antagonist, beta-blocker, ACE inhibitor or ARB, diuretics, and statin. Model 4 included model 3 and hemoglobin, eGFR, HDL, and LDL. We explored the potential influence of unmeasured confounders on our risk estimates using *E*-value analysis to determine how solid and imbalanced a confounding effect would need to be to alter the direction of findings (21). We tested for interactions of age, sex, diabetes mellitus, hypertension, prior cardiovascular disease, hyperlipidemia, and malnutrition (serum albumin <3.6 g/dL).

### Sensitivity Analysis

To further explore the potential effect of imbalanced confounders, we performed propensity score-matched sensitivity analyses that compared low ratio with moderate ratio and high ratio with moderate ratio by using a greedy matching algorithm with a caliper width of 0.2 of the propensity score (22). To minimize the potential for reverse causation, we conducted analyses that excluded patients with prior cardiovascular disease or those deaths in the first years of follow-up. When considering a transfer to hemodialysis, receiving renal transplantation, transfer to other centers, and loss of follow-up as competing risks for all-cause mortality, we further analyzed the association between albumin to total cholesterol ratio and all-cause mortality using the Gray test. Missing data for serum albumin (*n* = 23), total cholesterol (35), or any other explanatory variables (*n* = 121) at the start of PD were replaced by the most recent available values by checking patients’ medical records of receiving the first PD procedure. All analyses were conducted with the use of Stata 15.1. statistical software (StataCorp, College Station, TX, United States).

## Results

### Baseline Characteristics

We excluded 55 patients aged <18 years, 62 patients with <3 months of follow-up, and 2 patients with serum albumin ≥6.0 g/dL. Thus, 3447 eligible patients were finally included in the present study (Supplementary Figure 1).

**FIGURE 1:**
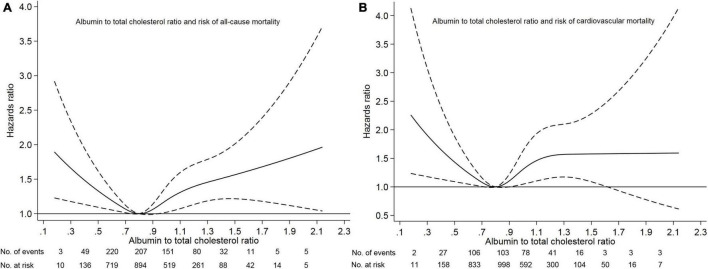
Association of albumin to total cholesterol ratio with risk of mortality. Panel **(A)** showed a restricted-cubic-spline plot of the association between albumin to total cholesterol ratio and all-cause mortality. Panel **(B)** showed a restricted cubic-spline plot of the association of albumin to total cholesterol ratio and cardiovascular mortality. All plots were adjusted for age, sex, body mass index, systolic blood pressure, current smoking, alcohol intake, comorbid conditions, medication use, laboratory measurements. Dashed lines indicate 95% confidence intervals. The median albumin to total cholesterol ratio (0.80) was the reference standard, indicated by the gray line.

Of 3447 patients with a mean age of 49.7 years, 1795 (52.1%) were male sex, 662 (19.2%) had diabetes mellitus, 2415 (70.1%) had hypertension, 368 (10.7%) had a prior cardiovascular disease, and 596 (17.3%) had hyperlipidemia. The median albumin to total cholesterol ratio was 0.80 (range, 0.11–2.14). Based on our restricted-cubic-spline plots for the primary outcome, we selected a level of 0.77–0.82 as the reference category for the albumin to total cholesterol ratio (Figure 1). According to the albumin to total cholesterol ratio, the baseline characteristics of patients were shown in Table 1. The low group had low levels of serum albumin and high levels of total cholesterol, whereas the high group had high levels of serum albumin and low levels of total cholesterol. Compared with the moderate group, the high group was younger and less likely to have diabetes mellitus, had a history of cardiovascular disease, gastrointestinal bleeding, hyperlipidemia, taking calcium antagonist, beta-blocker, ACE inhibitor or ARB, and diuretics, and had lower systolic blood pressure, hemoglobin, eGFR, HDL, and LDL. In contrast, the low group was more likely to have diabetes mellitus and hyperlipidemia.

**TABLE 1:** Baseline patient characteristics by categories of albumin to total cholesterol ratio.

		Albumin to total cholesterol ratio			
		
	Overall	Low ratio (<0.77)	Moderate ratio (0.77–0.82)	High ratio (>0.82)	*P*-value
Number of patients, *n*	3447	1523	364	1560	
Albumin to total cholesterol ratio	0.80 ± 0.30	0.62 ± 0.10	0.79 ± 0.02	1.07 ± 0.22	
Albumin (g/dL)	3.45 ± 0.53	3.19 ± 0.48	3.47 ± 0.44	3.67 ± 0.45	<0.001
Total cholesterol	4.40 ± 1.20	5.25 ± 1.04	4.38 ± 0.55	3.53 ± 0.68	<0.001
Age (years)	49.7 ± 14.5	50.8 ± 14.6	50.6 ± 14.2	48.4 ± 14.3	<0.001
Male sex, *n* (%)	1795 (52.1%)	781 (51.3%)	192 (52.7%)	822 (52.7%)	0.708
Body mass index (kg/m^2^)	22.3 ± 3.2	22.5 ± 3.3	22.4 ± 3.1	22.2 ± 3.2	0.088
Current smoker, *n* (%)	342 (9.9%)	138 (9.1%)	36 (9.9%)	168 (10.8%)	0.284
Current alcohol use, *n* (%)	126 (3.7%)	47 (3.1%)	11 (3.0%)	68 (4.4%)	0.135
Systolic blood pressure (mmHg)	147.5 ± 22.9	150.4 ± 23.5	148.4 ± 23.1	144.5 ± 21.7	<0.001
Diastolic blood pressure (mmHg)	87.0 ± 14.0	87.4 ± 14.1	87.1 ± 14.4	86.6 ± 13.8	0.319
**Comorbidities, *n* (%)**					
Diabetes mellitus	662 (19.2%)	385 (25.3%)	74 (20.3%)	203 (13.0%)	<0.001
Hypertension	2415 (70.1%)	1073 (70.5%)	257 (70.6%)	1085 (69.6%)	0.923
Prior cardiovascular disease	368 (10.7%)	226 (14.8%)	45 (12.4%)	97 (6.2%)	<0.001
COPD	31 (0.9%)	12 (0.8%)	3 (0.8%)	16 (1.0%)	0.773
Gastrointestinal bleeding	96 (2.8%)	52 (3.4%)	20 (5.5%)	24 (1.5%)	<0.001
Hyperlipidemia	596 (17.3%)	370 (24.3%)	62 (17.0%)	164 (10.5%)	<0.001
**Medication use, *n* (%)**					
Calcium antagonist	2202 (63.9%)	990 (65.0%)	253 (69.4%)	959 (61.5%)	<0.001
Beta-blocker	1293 (37.5%)	623 (40.9%)	147 (40.4%)	523 (33.5%)	<0.001
ACE inhibitor or ARB	939 (27.2%)	446 (29.3%)	108 (29.7%)	385 (24.7%)	0.009
Diuretics	540 (15.7%)	289 (19.0%)	59 (16.2%)	192 (12.3%)	<0.001
Statins	508 (14.7%)	249 (16.3%)	45 (12.4%)	214 (13.7%)	0.048
**Laboratory measurements**					
Hemoglobin (g/L)	87.6 ± 19.7	90.2 ± 19.5	88.8 ± 19.1	84.9 ± 19.8	<0.001
eGFR (mL/min × 1.73 m^2^)	7.2 ± 3.8	7.6 ± 4.0	7.4 ± 4.3	6.7 ± 3.4	<0.001
HDL (mmol/L)	1.1 ± 0.4	1.2 ± 0.4	1.2 ± 0.5	1.0 ± 0.3	<0.001
LDL (mmol/L)	2.6 ± 0.9	2.7 ± 0.9	2.7 ± 0.8	2.4 ± 0.8	<0.001

*COPD, chronic obstructive pulmonary disease; ACEI, angiotensin-converting enzyme inhibitor; ARB, angiotensin receptor blocker; eGFR, estimated glomerular filtration rate; HDL, high-density lipoprotein; LDL, low-density lipoprotein.*

### Albumin to Total Cholesterol Ratio and Mortality

During the median of 39.3 (range, 3.1–181.5) months of follow-up, 762 (22.1%) patients died, 466 (13.5%) patients transferred to hemodialysis, 229 (6.6%) patients received renal transplantation, 434 (12.6%) patients transferred to other dialysis centers, and 58 (1.7%) patients were loss to follow-up. Of 762 deaths, 382 (50.1%) deaths were due to cardiovascular disease, 135 (17.8%) deaths due to infectious disease, 72 (9.4%) deaths due to gastrointestinal bleeding, 14 (1.8%) deaths due to malignancy, 66 (8.7%) deaths due to other reasons, and 93 (12.2%) deaths due to unknown reasons. Deaths occurred in 356 (56.5/1000 person-years), 56 (38.3/1000 person-years), and 350 (57.0/1000 person-years) patients in those <0.77, 0.77–0.82, and >0.82 patients, respectively (Table 2).

**TABLE 2:** Incidence rate of death according to albumin to total cholesterol ratio.

	Albumin to total cholesterol ratio			
	
Outcomes	All levels	Low ratio (<0.77)	Moderate ratio (0.77–0.82)	High ratio (>0.82)
**All-cause mortality**				
Deaths, *n*	762	356	56	350
Deaths, per 1000 person-years	54.8	56.5	38.3	57.0
**Cardiovascular mortality**				
Deaths, *n*	382	174	22	186
Deaths, per 1000 person-years	27.5	27.6	15.0	30.3

*The incidence rate was calculated by dividing the proportion of events by the total effective observation time in the risk, which is converted to the number of episodes per 1000 years.*

**FIGURE 2:**
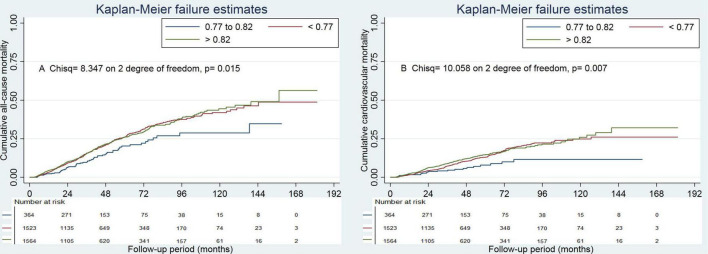
Cumulative mortality by categories of albumin to total cholesterol ratio. Panel **(A)** showed cumulative all-cause mortality by categories of albumin to total cholesterol ratio. Panel **(B)** showed cumulative cardiovascular mortality by categories of albumin to total cholesterol ratio.

Survival analyses showed that the albumin to total cholesterol ratio of 0.77–0.82 had the lowest cumulative all-cause and cardiovascular mortality (Figure 2). As compared with an albumin to total cholesterol ratio of 0.77–0.82 (the reference category), an albumin to total cholesterol ratio of >0.82 was associated with increased risks of all-cause mortality [hazards ratio (HR), 1.54; 95% confidence interval (CI), 1.16–2.05; *E*-value = 2.45] and cardiovascular mortality (HR, 2.10 95% CI, 1.35–3.29; *E*-value = 3.46) on multivariable analysis (Tables 3, 4 and Figure 1). Plus, as compared with the reference range, an albumin to total cholesterol ratio (<0.77) was also associated with increased risks of all-cause mortality (HR, 1.46; 95% CI, 1.10–1.94; *E*-value = 2.28) and cardiovascular mortality (HR, 1.78; 95% CI, 1.14–2.78; *E*-value = 3.62) on multivariable analysis (Tables 3, 4 and Figure 1).

**TABLE 3:** Association between albumin to total cholesterol ratio and all-cause mortality*.

	HR (95% CI) by albumin to total cholesterol ratio		
	
	Low ratio (<0.77)	Moderate ratio (0.77–0.82)	High ratio (>0.82)
All-cause mortality, *n* (%)	356 (23.4%)	56 (15.4%)	351 (22.4%)
Univariate	1.48 (1.11–1.96)	1.0	1.50 (1.13–1.99)
Multivariable	1.46 (1.10–1.94)	1.0	1.54 (1.16–2.05)
Without prior cardiovascular disease	1.39 (1.03–1.88)	1.0	1.50 (1.11–2.03)
Without deaths during the first year of follow-up	1.45 (1.08–1.97)	1.0	1.46 (1.07–2.00)

*Unless stated, model adjusted for age, sex, body mass index, current smoking, alcohol intake, systolic blood pressure, comorbid conditions, medication use, laboratory measurements.

**TABLE 4:** Association between albumin to total cholesterol ratio and cardiovascular mortality*.

	HR (95% CI) by albumin to total cholesterol ratio		
	
	Low ratio (<0.77)	Moderate ratio (0.77–0.82)	High ratio (>0.82)
Cardiovascular mortality, *n* (%)	174 (11.4%)	22 (6.0%)	186 (11.9%)
Univariate	1.83 (1.18–2.86)	1.0	2.02 (1.30–3.14)
Multivariable	1.78 (1.14–2.78)	1.0	2.10 (1.35–3.29)
Without prior cardiovascular disease	1.81 (1.12–2.92)	1.0	2.09 (1.30–3.38)
Without deaths during the first year of follow-up	2.11 (1.26–3.53)	1.0	2.41 (1.43–4.06)

*Unless stated, model adjusted for age, sex, body mass index, current smoking, alcohol intake, systolic blood pressure, comorbid conditions, medication use, laboratory measurements.

### Subgroup Analysis

There were no significant subgroup interactions (Supplementary Tables 1, 2). Similar findings were observed in subgroup analyses.

### Sensitivity Analysis

The exclusion of patients with prior cardiovascular disease or those who died in the first year of follow-up did not materially affect the results from the albumin to total cholesterol ratio analyses (Tables 3, 4).

When considering a transfer to hemodialysis, receiving renal transplantation, transfer to other centers, as well as loss of follow-up as competing risks for all-cause mortality, we found that compared with the moderate group, the high and low groups were associated with 1.50 (95% CI, 1.13–1.99; *E*-value = 2.37) and 1.48 (95% CI, 1.11–1.96; *E*-value = 2.32) times higher risk of all-cause mortality, respectively (Supplementary Table 3).

In a propensity-score-matched analysis that compared with a moderate level (0.77–0.82), high albumin to total cholesterol ratio (>0.82) was associated with increased risks of all-cause mortality (HR, 1.92; 95% CI, 1.30–2.82) and cardiovascular mortality (HR, 2.43; 95% CI, 1.37–4.30; Table 5). In a similar analysis that as compared with a moderate level, low albumin to total cholesterol ratio (<0.77) was associated with increased risks of all-cause mortality (HR, 1.57; 95% CI, 1.08–2.30) and cardiovascular mortality (HR, 1.97; 95% CI, 1.15–3.39; Table 5).

**TABLE 5:** Propensity score-based matched analysis for comparison of low to moderate albumin to total cholesterol ratio and high to moderate albumin to total cholesterol ratio.

	Albumin to total cholesterol ratio		
	
	Low vs. moderate	Moderate	High vs. moderate
All-cause mortality, *n* (%)	81 (22.3%)	56 (15.4%)	97 (26.6%)
HR (95%)	1.57 (1.08–2.30)	1.0	1.92 (1.30–2.82)
Cardiovascular mortality, *n* (%)	41 (11.3%)	22 (6.0%)	51 (14.0%)
HR (95%)	1.97 (1.15–3.39)	1.0	2.43 (1.37–4.30)

*Analysis: We developed a multivariable logistic regression model to estimate propensity scores for low (<0.77) and high (>0.82) albumin to total cholesterol ratio, using the following covariates: age, sex, body mass index, diabetes mellitus, prior cardiovascular disease, and hypertension. Each patient within these two groups was assigned a propensity score. We then matched patients with low albumin to total cholesterol ratio (<0.77) to those with moderate albumin to total cholesterol ratio (0.77–0.82), and matched those with high albumin to total cholesterol ratio (>0.82) to those with moderate albumin to total cholesterol ratio, by using a greedy matching algorithm with a caliper width of 0.2 of the propensity score. Baseline patient characteristics between low and moderate albumin to total cholesterol ratio and between high and moderate albumin to total cholesterol ratio were well balanced (data not shown).*

## Discussion

In this large, retrospective cohort study, we found the lowest risk of death among patients with albumin to total cholesterol ratio between 0.77 and 0.82. Both higher and lower albumin levels to total cholesterol ratio were associated with increased risk, resulting in a U-shaped association curve. Notably, although patients with higher albumin to cholesterol ratio at baseline were younger and less likely to present with comorbidities than those with a moderate ratio, they remained to be at higher risk of death, suggesting that lower levels of total cholesterol may be more strongly associated with a poorer prognosis in PD patients.

As we know, low serum albumin is associated with high mortality in patients with end-stage renal disease (ESRD) on chronic dialysis. A prospective cohort study of 680 consecutive CAPD patients in 14 centers in Canada and the United States reported that each 1 g/dL increase in the plasma albumin concentration was associated with a decrease in the relative risk of death by 6% (23). Another previous study with more than 8000 hemodialysis patients having low albumin also reported that of the laboratory variables, low serum albumin less than 4.0 g/dL was most highly associated with death probability (14). Similar results also were observed in other studies (11–13). There is a graded relationship between total cholesterol concentration and coronary risk among general patients, and the absolute risk is greater in those with prior manifestations of coronary heart disease (24).

However, the relationship is not the same in dialysis patients. A prospective cohort of 1167 chronic hemodialysis patients found that hypocholesterolemia (<3.6 mmol/L) was an independent predictor of death in hemodialysis patients (15). Serum total cholesterol was a significant predictor of death with an adjusted HR (95% CI) was 0.94 (0.89–0.99). Similarly, other studies also found similar results (14, 16, 17). Thus, there is pending a question: among dialysis patients combining lower serum albumin and higher serum total cholesterol, how is the prognosis? More importantly, many dialysis patients have the conditions above in clinical settings. Therefore, we need a variable combining serum albumin and total cholesterol to examine the association. Based on previous studies (18, 25), we considered the albumin to total cholesterol ratio an optimal variable.

An early study reported that the total cholesterol to albumin ratio could be used as an alternative parameter in predicting the risk of cardiovascular disease in 30 patients with a history of cardiovascular disease, with 5.0 as a cutoff value (18). Another early study found that replacing total cholesterol to HDL with total cholesterol to albumin in general outpatients was likely to weaken the relation with coronary heart disease (25). However, in recent decades, no study has further examined the association between serum albumin to total cholesterol ratio and prognosis in any specific population. In the present study, we first reported the association between albumin to total cholesterol ratio and mortality in PD patients. We found a U-shaped association between albumin to total cholesterol ratio and mortality. Our results were robust because we showed consistent results, even after adjusting for confounding factors or balancing baseline characteristics using propensity-score-matched analysis or sensitivity analysis.

Previous studies have reported that lower serum albumin is associated with a higher risk of mortality (11–13), and higher cholesterol levels have been consistently associated with lower mortality in dialysis patients (14–17). Thus, lower albumin to total cholesterol ratio was associated with an increased risk of mortality, suggesting that patients with lower albumin and higher total cholesterol may have a poorer prognosis. These findings suggested that the effect power of lower albumin levels on mortality was more potent than that of higher total cholesterol on mortality. Similarity, higher albumin to total cholesterol was also associated with increased risk mortality, suggesting that patients with higher albumin and lower total cholesterol may have a poorer prognosis. These findings also suggested that the effect power of lower total cholesterol on mortality was more potent than that of higher albumin on mortality. Needless to say, a large prospective study needs to be conducted to identify our findings and speculation.

An earlier study of 823 dialysis patients reported hypercholesterolemia is a risk factor for all-cause and cardiovascular mortality. This association was masked among those with malnutrition (26). In this study, an inverse association of cholesterol levels with all-cause mortality and a U-shaped relationship with cardiovascular mortality were observed in malnutrition, whereas a strong, graded, positive association of total cholesterol with all-cause and cardiovascular mortality in the absence of malnutrition. In the present study, we did not find that malnutrition masked the association of albumin to total cholesterol ratio with all-cause and cardiovascular mortality.

Strengths include a large number of patients, the high integrity of the data, and the availability of detailed covariates that can be adjusted for a wide range of potential confounders. In this study, several limitations should be acknowledged, and findings should be interpreted with these carefully. First, because this was an observational study, the possibility of residual confounding from unmeasured variables was a potential limitation. We used the *E*-value analysis to examine the possibility. For example, even a substantial confounding effect (HR ≥2.45) would require a significant imbalance between the high and moderate albumin-to-total cholesterol ratio categories to result in an adjusted HR below 1.0. Weaker confounders cannot do this. Our study had adjusted for these traditional strong confounders such as age, current smoker, current alcohol use, diabetes mellitus, prior cardiovascular disease, and hypertension. In addition, we used propensity score-matched analyses to actively control confounding variables. Second, we only analyzed the albumin to total cholesterol ratio at the initiation of PD. The single albumin to total cholesterol ratio measurement at baseline may have underestimated the association due to regression dilution bias (27). Notably, regression dilution bias may in part lead to over-adjustment (28). Nonetheless, serial measurements could provide more information than a single measurement. Third, missing values were replaced by the most recent available deals of the first PD, not using multiple imputations. Although multiple imputations can randomly fill in these missing values, the latest available value may represent the clinical status more appropriately. Lastly, all eligible patients were from China, suggesting that the findings may lack general applicability to other ethnic groups. In addition, automated PD may lead to a significant decline in serum albumin and higher total cholesterol levels (29, 30). Thus our results were also not generalizable in patients on a cycler.

In summary, lower or higher albumin to total cholesterol ratio before the first PD was associated with increased mortality, resulting in a U-shaped association. Although the underlying mechanisms are unclear, using an inexpensive and readily available biochemical biomarker, serum albumin to total cholesterol ratio, may improve the stratification risk of mortality in PD patients.

## Data Availability Statement

The raw data supporting the conclusions of this article will be made available by the authors, without undue reservation.

## Ethics Statement

The studies involving human participants were reviewed and approved by the Ethics Committee of The First Affiliated Hospital of Zhengzhou University, Zhengzhou, China; The Ethics Committee of The First Affiliated Hospital of Nanchang University, Nanchang, China; The Ethics Committee of Jiujiang No. 1 People’s Hospital, Jiujiang, China; The Ethics Committee of Zhujiang Hospital of Southern Medical University, Guangzhou, China; The Ethics Committee of The Second Affiliated Hospital of Guangzhou Medical University, Guangzhou, China. Written informed consent for participation was not required for this study in accordance with the national legislation and the institutional requirements.

## Author Contributions

XFW and JM: conceptualization. XFW, JW, and JM: methodology. XFW, JM, and LZ: software. JW: validation. XYW and XZ: formal analysis and investigation. XYW, XZ, FP, YW, and XF: resources. NW: data curation. JM: writing – original draft preparation. XFW and JW: writing – review and editing. All authors contributed to the article and approved the submitted version.

## Conflict of Interest

The authors declare that the research was conducted in the absence of any commercial or financial relationships that could be construed as a potential conflict of interest.

## Publisher’s Note

All claims expressed in this article are solely those of the authors and do not necessarily represent those of their affiliated organizations, or those of the publisher, the editors and the reviewers. Any product that may be evaluated in this article, or claim that may be made by its manufacturer, is not guaranteed or endorsed by the publisher.
