# Supplementary material for: Albumin to Total Cholesterol Ratio and Mortality in Peritoneal Dialysis
Source: Front Med (Lausanne). 2022 Jun 9;9:896443. doi: 10.3389/fmed.2022.896443 (PMC9218528; doi:10.3389/fmed.2022.896443)
Supplement: Supplementary file 1 [file Table_1.DOCX]

**Albumin to Total Cholesterol Ratio and Mortality in Peritoneal Dialysis**

**Supplementary Appendix**

**Materials & Methods**

**Study Design and Participants**

***Peritoneal dialysis centers of five tertiary hospitals***

The First Affiliated Hospital of Zhengzhou University, Zhengzhou, China.

The First Affiliated Hospital of Nanchang University, Nanchang, China.

Jiujiang No. 1 People’s Hospital, Jiujiang, China.

Zhujiang Hospital of Southern Medical University, Guangzhou, China.

The Second Affiliated Hospital of Guangzhou Medical University, Guangzhou, China.

***Definitions***

Cardiovascular mortality included death associated with an acute myocardial ischemic event, heart failure, hemorrhagic or thromboembolic stroke, malignant arrhythmia, and sudden cardiac death, based on the International Classification of Diseases Clinical Modification, 9th Revision. Sudden cardiac death is defined as unexpected, nontraumatic death occurring within 1hour of the onset of new or worsening symptoms (witnessed arrest) or, if unwitnessed, within 24 hours of last being seen alive^1^. Hypertension was defined as systolic blood pressure > 140 mmHg, diastolic blood pressure > 90 mmHg, or the use of antihypertensive medications. Diabetes mellitus was defined as a history of diabetes mellitus. Hyperlipidemia was defined as (1) serum cholesterol levels ≥4.7 mmol/L, (2) triglyceride levels ≥2.3mmol/L, or (3) low density lipoprotein levels ≥4.1mmol/L^2^. Patients who meet one of these three items are defined as having hyperlipidemia. Current smoking was defined as at least one cigarette a day, and current alcohol consumption was defined as > 20 g of ethanol a day^3^. Chronic obstructive pulmonary disease is characterized by persistent respiratory symptoms and progressive airflow obstruction^4^. Gastrointestinal bleeding includes upper and lower gastrointestinal bleeding^5^. The Chronic Kidney Disease Epidemiology Collaboration equation was used to calculate eGFR^6^.

**Missing data**

In China, the patient must receive the first dialysis procedure in the hospital. To obtain missing patients’ demographic characteristics, comorbid conditions, medication use, and laboratory variables, we had checked the medical records of receiving the first PD procedure. Thus, missing data for serum albumin (n=23), total cholesterol (35), or any other explanatory variables (n=121) at the start of PD were replaced by the most recent available values by checking patients’ medical records of receiving the first PD procedure.

**Table S1. Association of albumin to total cholesterol ratio with all-cause mortality in subgroups.**

|  | Albumin to total cholesterol ratio | | |  |
| --- | --- | --- | --- | --- |
|  | Low ratio (< 0.77) | Moderate ratio (0.77-0.82) | High ratio (> 0.82) | P-interaction |
| <65 years | 1.67 (1.08-2.05) | 1.0 | 1.54 (1.12-2.12) | 0.976 |
| ≥65 years | 1.38 (0.74-2.57) | 1.0 | 1.43 (0.76-2.69) |  |
| Male | 1.40 (0.95-2.07) | 1.0 | 1.42 (0.96-2.09) | 0.636 |
| Female | 1.54 (1.02-2.33) | 1.0 | 1.63 (1.08-2.47) |  |
| Hypertension | 1.38 (0.99-1.92) | 1.0 | 1.44 (1.03-1.99) | 0.929 |
| No hypertension | 1.73 (0.99-3.02) | 1.0 | 1.59 (0.91-2.79) |  |
| Diabetes mellitus | 1.33 (0.72-2.44) | 1.0 | 1.74 (0.93-3.26) | 0.903 |
| No diabetes mellitus | 1.52 (1.10-2.09) | 1.0 | 1.48 (1.08-2.04) |  |
| Prior cardiovascular disease | 1.82 (0.77-4.30) | 1.0 | 1.59 (0.63-3.98) | 0.908 |
| No prior cardiovascular disease | 1.41 (1.04-1.90) | 1.0 | 1.46 (1.08-1.97) |  |
| Hyperlipidemia | 2.66 (1.08-6.57) | 1.0 | 1.98 (0.76-5.12) | 0.261 |
| No hyperlipidemia | 1.34 (0.99-1.81) | 1.0 | 1.42 (1.06-1.91) |  |
| Albumin <3.6 g/dL | 1.28 (0.91-1.80) | 1.0 | 1.49 (1.04-2.12) | 0.893 |
| Albumin >=3.6 g/dL | 2.03 (1.20-3.41) | 1.0 | 1.69 (1.04-2.74) |  |

All analyses adjusted for age, sex, body mass index, current smoker, current alcohol use, systolic blood pressure, comorbidities, medication use, and lab measurements.

**Table S2. Association of albumin to total cholesterol ratio with cardiovascular mortality in subgroups.**

|  | Albumin to total cholesterol ratio | | |  |
| --- | --- | --- | --- | --- |
|  | Low ratio (< 0.77) | Moderate ratio (0.77-0.82) | High ratio (> 0.82) | P-interaction |
| <65 years | 1.62 (0.99-2.61) | 1.0 | 1.97 (1.22-3.16) | 0.739 |
| ≥65 years | 3.08 (0.95-9.96) | 1.0 | 2.68 (0.81-8.86) |  |
| Male | 1.78 (0.99-3.17) | 1.0 | 1.82 (1.02-3.24) | 0.415 |
| Female | 1.89 (0.95-3.78) | 1.0 | 2.38 (1.20-4.73) |  |
| Hypertension | 1.59 (0.95-2.64) | 1.0 | 1.94 (1.17-3.21) | 0.911 |
| No hypertension | 2.44 (0.98-6.08) | 1.0 | 2.38 (0.95-5.95) |  |
| Diabetes mellitus | 1.26 (0.57-2.80) | 1.0 | 1.76 (0.77-4.00) | 0.874 |
| No diabetes mellitus | 2.07 (1.21-3.55) | 1.0 | 2.25 (1.32-3.82) |  |
| Prior cardiovascular disease | 1.65 (1.03-1.94) | 1.0 | 1.85 (1.01-1.96) | 0.602 |
| No prior cardiovascular disease | 1.84 (1.14-2.96) | 1.0 | 2.02 (1.26-3.26) |  |
| Hyperlipidemia | 2.17 (0.67-7.02) | 1.0 | 1.83 (0.53-6.37) | 0.326 |
| No hyperlipidemia | 1.71 (1.05-2.76) | 1.0 | 2.03 (1.26-3.27) |  |
| Albumin <3.6 g/dL | 1.58 (0.93-2.69) | 1.0 | 2.01 (1.16-2.69) | 0.631 |
| Albumin >=3.6 g/dL | 2.48 (1.10-5.62) | 1.0 | 2.34 (1.09-5.74) |  |

All analyses adjusted for age, sex, body mass index, current smoker, current alcohol use, systolic blood pressure, comorbidities, medication use, and lab measurements.

**Table S3. Association of albumin to total cholesterol ratio with mortality using competing risk model.**

|  | Albumin to total cholesterol ratio | | |
| --- | --- | --- | --- |
|  | Low ratio (< 0.77) | Moderate ratio (0.77-0.82) | High ratio (> 0.82) |
| Hazards ratio (95%CI) | 1.48 (1.12-1.96) | 1.0 | 1.50 (1.13-1.99) |

Transfer to hemodialysis, receiving renal transplantation, transfer to other centers, and loss of follow-up were competing risks for all-cause mortality.

All analyses adjusted for age, sex, body mass index, current smoker, current alcohol use, systolic blood pressure, comorbidities, medication use, and laboratory measurements, using the Gray test.

**
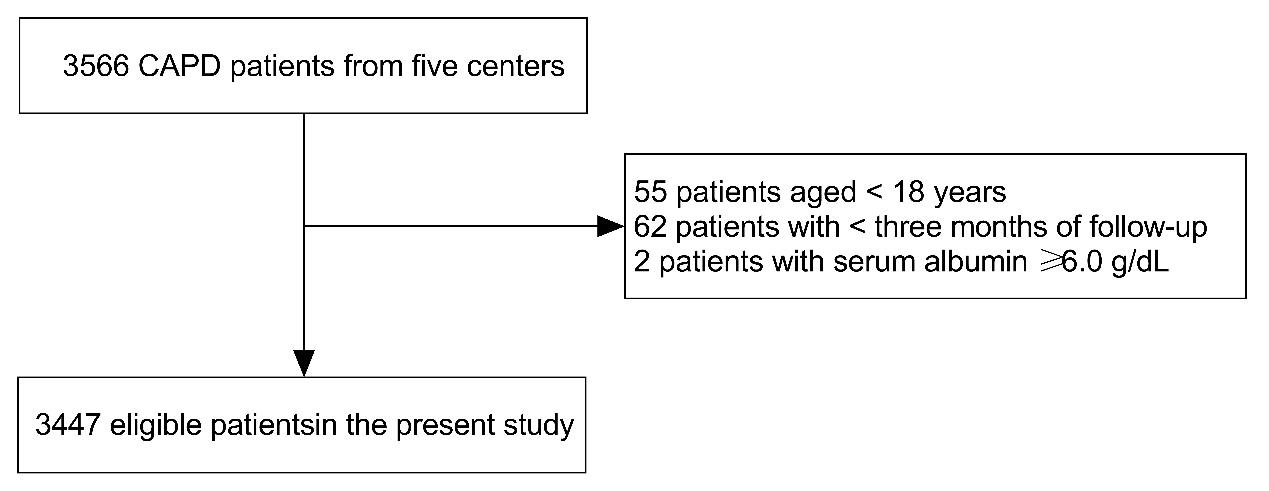
**

**Figure S1. The consort flow diagram**

**CAPD, continuous ambulatory peritoneal dialysis.**

**Reference**

1. European Heart Rhythm A, Heart Rhythm S, Zipes DP, et al. ACC/AHA/ESC 2006 guidelines for management of patients with ventricular arrhythmias and the prevention of sudden cardiac death: a report of the American College of Cardiology/American Heart Association Task Force and the European Society of Cardiology Committee for Practice Guidelines (Writing Committee to Develop Guidelines for Management of Patients With Ventricular Arrhythmias and the Prevention of Sudden Cardiac Death). *J Am Coll Cardiol.* 2006;48(5):e247-346.

2. Joint committee issued Chinese guideline for the management of dyslipidemia in a. [2016 Chinese guideline for the management of dyslipidemia in adults]. *Zhonghua Xin Xue Guan Bing Za Zhi.* 2016;44(10):833-853.

3. Tu W, Wu J, Jian G, et al. Asymptomatic hyperuricemia and incident stroke in elderly Chinese patients without comorbidities. *Eur J Clin Nutr.* 2019;73(10):1392-1402.

4. Labaki WW, Rosenberg SR. Chronic Obstructive Pulmonary Disease. *Ann Intern Med.* 2020;173(3):ITC17-ITC32.

5. Marek TA. Gastrointestinal bleeding. *Endoscopy.* 2011;43(11):971-977.

6. Zhang L, Wang F, Wang L, et al. Prevalence of chronic kidney disease in China: a cross-sectional survey. *Lancet.* 2012;379(9818):815-822.
